# Clinical course and risk factors for development and progression of interstitial lung disease in primary Sjögren’s syndrome

**DOI:** 10.1038/s41598-023-35608-4

**Published:** 2023-06-06

**Authors:** Kyung-Ann Lee, Bo Da Nam, Jung Hwa Hwang, Hyun-Sook Kim

**Affiliations:** 1 Division of Rheumatology, Department of Internal Medicine, Soonchunhyang University Seoul Hospital, 59 Daesagwan-ro, Yongsan-gu, Seoul, 04401 South Korea; 2 Department of Radiology, Soonchunhyang University Seoul Hospital, Soonchunhyang University School of Medicine, Seoul, South Korea

**Keywords:** Diseases, Medical research, Rheumatology, Risk factors

## Abstract

This single-center, retrospective study aimed to investigate the course and prognostic factors of patients with primary Sjögren syndrome-associated interstitial lung disease (pSS-ILD). We included 120 pSS patients who underwent at least two high-resolution computed tomography (HRCT) scans between 2013 and 2021. Clinical symptoms, laboratory data, HRCT findings, and pulmonary function test results were collected. Two thoracic radiologists reviewed the HRCT findings. In patients with pSS without ILD at baseline (n = 81), no development of ILD was found on follow-up (median, 2.8 years). In patients with pSS-ILD (n = 39), total disease extent, extent of coarse reticulation, and traction bronchiectasis increased on HRCT, whereas the extent of ground glass opacity (GGO) decreased at follow-up (median, 3.2 years) (each *p* < 0.001). In progressive group of pSS-ILD (48.7%), the extent of coarse reticulation and coarseness score of fibrosis were increased at follow-up (*p* < 0.05). Usual interstitial pneumonia pattern on CT (OR, 15.237) and follow-up duration (OR, 1.403) were independent risk factors for disease progression in patients with pSS-ILD. In both progressive and non-progressive pSS-ILD, GGO decreased, whereas the extent of fibrosis increased even after treatment with glucocorticoid and/or immunosuppressants. In conclusion, progression occurred in approximately half of the pSS-ILD patients with slow gradual deterioration. Our study identified a definite group of progressive pSS-ILD who did not respond to current anti-inflammatory treatment.

## Introduction

Primary Sjögren’s syndrome (pSS) is a chronic systemic inflammatory disease characterized by impairment of the lacrimal and salivary glands. Various extraglandular involvements, including the lung, nerve, kidney, skin, musculoskeletal, and hematopoietic systems have been reported^1^. Interstitial lung disease (ILD) is the most frequent pulmonary manifestation in patients with pSS, and ILD can be found in approximately 10–20% of pSS patients. Older age, male sex, smoking, positivity for anti-nuclear antibody, and longer disease duration are associated with the development of pSS-associated ILD (pSS-ILD). Interstitial lung disease can result in significant morbidity and mortality in patients with pSS^2,3^.

The onset and course of ILD vary in patients with pSS. Interstitial lung disease can appear in the late course of pSS, concomitant with other manifestations of the disease, or precede the onset of pSS^4^. In a series of 21 patients with pSS-ILD, Roca et al. showed diverse courses of ILD as follows: improvement (15.8%), stabilization (47.4%), or deterioration (36.8%)^5^. In 49 patients with pSS-ILD who repeated pulmonary function tests (PTFs) at six months from the baseline, 20.4% of patients showed progression^6^. In these studies, forced vital capacity (FVC) and diffusing capacity for carbon monoxide (DLCO) were used as indicators of ILD deterioration. However, changes in ILD based on high-resolution computed tomography (HRCT) findings were not investigated.

The severity and progression of ILD are key factors to consider when making treatment decisions for patients with pSS-ILD^7^. Serial PFTs are a simple, non-invasive method for monitoring disease progression in patients with ILD. However, intra-patient visit-to-visit variability in FVC measurements over time and a number of other factors affecting FVC results, such as comorbidities and aging make it difficult to assess the progression of ILDs^8,9^. Therefore, clinicians should determine ILD progression based on comprehensive evaluations, including worsening respiratory symptoms, PFTs, and chest imaging findings. High-resolution computed tomography is the most sensitive imaging tool and gold standard for the diagnosis of ILDs. Previous studies focusing on CT patterns showed that non-specific interstitial pneumonia (NSIP) was the most frequent pattern in pSS-ILD^10^, and the usual interstitial pneumonia (UIP) pattern was a determinant of progression and mortality^11^. Furthermore, HRCT can provide quantitative information on the extent and severity of the disease, as well as pattern analysis. To demonstrate the characteristics of disease progression and identify the risk factors for deterioration in patients with pSS-ILD, further larger studies with long-term clinical and radiographic follow-up are needed.

Therefore, we aimed to: (1) investigate the clinical and radiographic course based on HRCT findings in patients with pSS-ILD; and (2) determine the risk factors associated with pSS-ILD and disease progression in patients with pSS.

## Methods

### Study design and population

This single-center, retrospective, observational study enrolled patients with pSS who met the 2016 American College of Rheumatology/European League Against Rheumatism (ACR/EULAR) classification criteria and were aged ≥ 19 years at Soonchunhyang University Seoul Hospital between March 2013 and February 2021^12^. For inclusion, the patients should have undergone at least two HRCT scans at baseline and follow-up, with intervals of a minimum of six months. Patients with secondary SS or those who met the exclusion criteria according to the 2016 ACR/EULAR classification criteria^12^, incomplete clinical data, or a history of malignancy or radiation therapy were excluded.

This study was conducted in accordance with the Declaration of Helsinki and approved by the Institutional Review Board (IRB) of Soonchunhyang University Seoul Hospital (IRB Number:2020-07-033). The requirement for patient approval or informed consent was waived by the IRB of Soonchunhyang University Seoul Hospital, owing to the retrospective nature of the study and because the analysis used anonymous clinical data.

### Data collection

The following data were collected for all patients: respiratory symptoms, disease duration, smoking history, autoantibodies (anti-nuclear antibody, anti-SSA/Ro, anti-SSB/La, and rheumatoid factor), Schirmer’s test result (≤ 5 mm/5 min on at least one side was abnormal), whole unstimulated salivary flow rate test, ocular staining score, focus score, salivary gland ultrasound (SGUS) according to the Outcome Measures in Rheumatology (OMERACT) US scoring system^13^, Sjögren's Syndrome Disease Damage Index (SSDDI)^14^, FVC, and DLCO (corrected for hemoglobin). In the enrolled patients, a diagnosis of pSS-ILD was established based on HRCT findings. The FVC and DLCO data were obtained from the baseline (at initial diagnosis of pSS-ILD) to the most recent follow-up in patients with pSS-ILD. Pulmonary function test data were missing for patients with severe pSS-ILD who were unable to perform the test properly. Information regarding mortality and causes of death was obtained from a review of medical records. Data on treatment strategies for pSS-ILD were also collected. Treatment for pSS-ILD was decided according to the clinical judgement based on respiratory symptoms, spirometry, and images, due to lack of consensus guidelines for pSS-ILD.

Progression of pSS-ILD was defined when any of the following condition was present during the follow-up period: (1) a relative decline in FVC of ≥ 10% from baseline or in DLCO of ≥ 15%; (2) a relative decline in FVC of 5–9% and increased extent of fibrosis on HRCT; (3) a relative decline in FVC of 5–9% and worsening respiratory symptoms; and (4) worsened respiratory symptoms and increased extent of fibrosis on HRCT^15^. Accordingly, patients without these findings were designated as having non-progressive pSS-ILD.

### Image acquisition

A total of 120 patients underwent 249 HRCT scans (mean follow-up intervals, 51.1 ± 45.3 months) using two types of CT scanners (Discovery CT750 HD; GE Healthcare, Milwaukee, WI, USA; and SOMATOM Definition Edge; Siemens Medical Solutions, Erlangen, Germany). All images were obtained caudocranially from the lung base through the thoracic inlet level. Image acquisition was performed in inspiratory and expiratory supine positions and in the inspiratory prone position. None of the patients received intravenous injections of contrast medium for the CT study. Scanning parameters were 120 kVp and 90–170 mA. Spiral CT scans (beam width of 10–20 mm, beam pitch of 1.375–1.5) were obtained throughout the thorax, and the scan data were reconstructed with 1.0-mm section thickness at 5-mm intervals. The CT data were reconstructed using a sharp kernel algorithm.

### Analysis of HRCT

Two board-certified thoracic radiologists with 11 and 30 years of experience retrospectively reviewed the HRCT findings by consensus and were blinded to the clinical and PFT results. Lung fields were divided into five levels: (i) origin of great vessels; (ii) main carina; (iii) pulmonary venous confluence; (iv) halfway between the third to fifth section; and (v) immediately above the right hemidiaphragm^16^. The following features were reviewed: ground-glass opacities (GGOs), reticulations, honeycombing, and traction bronchiectasis (BE)^17^. High-resolution computed tomography variables for ILD analysis included: total disease extent; presence and extent of individual features (GGO, reticulation, and honeycombing); coarseness of fibrosis; severity of traction BE; diagnosis of HRCT pattern (definition for each HRCT variable is shown in Supplemental data S1)^16,18^. Among patients with pSS-ILD, nine underwent additional HRCT scans prior to treatment for pSS-ILD. Two radiologists reviewed these scans to evaluate the changes after treatment.

### Statistical analysis

Statistical analyses were performed using SPSS (version 22.0; SPSS Inc., Chicago, IL, USA) and GraphPad Prism 8.0.1 Windows. Continuous variables are expressed as mean (standard deviation) or median (Q1, Q3), and categorical variables are presented as frequencies and proportions. Intergroup comparisons were conducted using the Student’s t-test or Mann–Whitney U test for continuous variables, while categorical data were compared using the chi-square test or Fisher’s exact test. The Wilcoxon signed rank test was used to compare continuous variables between the groups. The average annual changes in FVC, DLCO, and HRCT scores (total disease extent, extent of GGO, fine and coarse reticulation, coarseness score of fibrosis, and score for traction BE) were calculated for each patient as (last score − first score)/(number of years between scores) and summarized as the median (IQR) change per year^19^. Univariate and multivariate logistic regression analyses were performed to identify the independent risk factors for presence and progression of ILD in the pSS patients, and odds ratios (ORs) and 95% confidence intervals (CIs) were calculated. In the multivariable analysis, parameters with *P* values ≤ 0.1 in the univariate analysis were adjusted. Multicollinearity diagnostic tests were performed using the variance inflation factor (VIF) before determining the final model. No multicollinearity was defined as VIFM < 10. Statistical significance was set at *P* < 0.05.

## Results

### Baseline characteristics

A total of 39 patients with pSS-ILD and 81 patients without pSS-ILD were included in this study (Supplementary Figure. S1). In a pSS-ILD group, ILD diagnosis on HRCT was an earlier manifestation than the clinical diagnosis of pSS in 13 (33.3%) patients (median, -1.45 [− 4.2, − 0.63] years). In the remaining patients with pSS-ILD, ILD later manifested after the clinical diagnosis of pSS (median 0.13 [0, 0.39] years).

 Patients without ILD underwent the first HRCT at a median of 0.05 (0.01, 0.12) years after the clinical diagnosis of pSS. Additionally, ILD was not newly observed in pSS-patients having no ILD at baseline during the follow-up period of median 2.8 (1.1, 4.1) years.

Patients with pSS-ILD were older, more likely to be smokers, had higher anti-Ro52 levels, lactate dehydrogenase (LDH), and SSDDI, and lower DLCO at baseline. The involvement of exocrine glands and organs other than the lung was not different between pSS patients with and without ILD (Table 1). In pSS patients with ILD, DLCO was significantly lower than in those without ILD (62.8% vs. 82.7%, *p* < 0.01).

**Table 1:** Clinical and laboratory characteristics of the study population.

	pSS with ILD (n = 39)	pSS without ILD (n = 81)	P-value
Age at the baseline CT, years	67.1 (12.4)	57.1 (10.2)	** < 0.00**^**a**^
Male, n (%)	7 (17.9)	6 (7.4)	0*.*115^c^
Smoking, n (%)	6 (15.4)	1 (1.2)	**0.005**^**c**^
Follow-up, years, median (IQR)	4.1 (2.3–5.5)	4.5 (2.9–5.4)	0.622^b^
Interval time between the baseline and last CT, years, median (IQR)	3.2 (1.4–5.0)	2.8 (1.1–4.1)	0.169^b^
Body mass index, kg/m^2^	23.1 (3.1)	23.3 (3.6)	0.731^**a**^
Abnormal Schirmer’s test, n (%)	23/23 (100)	58/66 (87.9)	0.106^d^
Unstimulated salivary flow rate, ml/minute	0.18 (0.21)	0.19 (0.20)	0.883^**a**^
Ocular staining score ≥ 5, n (%)	6/19 (31.6)	23/57 (40.3)	0.495^d^
Laboratory findings			
Positive anti-Ro60, n (%)	23 (58.9)	50 (61.7)	0.772^c^
Positive anti-Ro52, n (%)	25 (64.1)	39 (48.1)	0.072^c^
Positive anti-La/SSB, n (%)	5 (12.8)	15 (18.5)	0.433^c^
Anti-Ro60, median (IQR)	1 (0–3)	2 (0–3)	0.741^b^
Anti-Ro52, median (IQR)	3 (0–3)	0 (0–3)	**0.045**^b^
Anti-La/SSB, median (IQR)	0 (0–0)	0 (0–0)	0.468^b^
Rheumatoid factor, IU/ml (normal range 0–14)	23.7 (34.1)	48.9 (266.4)	0.632^**a**^
IgG, mg/dL	1607 (775)	1290 (745)	0.094^**a**^
LDH, U/L	231.7 (53.9)	195.0 (38.1)	** < 0.001**^**a**^
**Focus score,** median (IQR)	1 (1–2)	1 (1–2)	1.000^b^
**Focus score** ≥ 1, n (%)	30/31 (96.8)	66/69 (95.6)	**1.000**^c^
**Total SGUS OMERACT scores,** median (IQR)	4 (0–7.75)	4 (0–8)	0.866^b^
Organ involvement			
SSDDI, median (IQR)	4 (2–5)	2 (1–3)	** < 0.001**^b^
Arthritis, n (%)	7 (17.9)	12 (14.8)	0.660^c^
Purpura, n (%)	1 (2.5)	2 (2.4)	1.000^d^
Autoimmune hepatitis, n (%)	2 (5.1)	2 (2.4)	0.595^d^
Lymphoma, n (%)	1 (2.5)	2 (2.4)	1.000^d^
**FVC** at baseline, % predicted	83.2 (19.8)	83.0 (38.7)	0.962^**a**^
**DLCO** at baseline, % predicted	62.8 (82.7)	82.7 (14.1)	**0.001**^**a**^

Values are expressed as mean (standard deviation) unless otherwise stated.

Intergroup comparisons were conducted using the ^a^Student’s t-test or ^b^Mann–Whitney U test for continuous variables, while categorical data were compared using the ^c^chi-square test or ^d^Fisher’s exact test.

pSS: primary Sjogren’s syndrome, ILD: interstitial lung disease, CT: computed tomography, SGUS: salivary gland ultrasonography, OMERACT: Outcome Measures in Rheumatology Clinical Trials, SSDDI: Sjögren's Syndrome Disease Damage Index, FVC: Forced vital capacity, DLCO: Diffusing capacity for carbon monoxide.

Significant values are in bold.

### Risk factors for presence of pSS-ILD

In univariate logistic regression analysis, age (OR, 1.087 [95% CI 1.044–1.131]), smoking (OR 14.545 [95% CI 1.685–125.558]), LDH (OR, 1.018 [95% CI 1.008–1.028]), and baseline DLCO (0.911 [95% CI 0.875–0.949]) were significant predictors of pSS-ILD. Multivariate logistic regression analysis showed LDH (OR, 1.012 [95% CI 1.000–1.024]) and DLCO (OR, 0.922 [95% CI (0.886–0.961)] were independent predictors of the presence of ILD in patients with pSS (online supplemental Table S1).

### Longitudinal changes in HRCT, FVC, and DLCO in patients with pSS-ILD

In patients with pSS-ILD, the median interval between baseline and last follow-up HRCT scans was 3.2 (1.4, 5.0) years. According to the HRCT scores, the total disease extent, extent of coarse reticulation, coarseness score of fibrosis, and traction BE scores were significantly increased, whereas the extent of GGO was significantly decreased between the baseline and last follow-up period (each *p* < 0.001, Wilcoxon signed-rank test). The median annual changes in total disease extent, extent of GGO, fine and coarse reticulation, coarseness score of fibrosis, and scores for traction BE were 0.74 (0, 2.61), − 0.43 (− 0.98, − 0.59), 0.04 (− 0.61, 3.52), and 0.45 (0.0, 0.76), 0.45 (0.0, 0.76), and 0.38 (0.0, 0.79) %/year, respectively.

No significant changes in FVC (*p* = 0.402) or DLCO (*p* = 0.905) (Wilcoxon signed-rank test) were found during the median follow-up period of 3.1 years in total patients with pSS-ILD. Annual changes in FVC and DLCO were − 1.17 (− 3.2–0.89) %/year, and 0.21 (− 1.45–1.59)%/year, respectively.

### Comparison of clinical and pulmonary functional findings between progressive and non-progressive pSS-ILD

Table 2 shows the clinical, laboratory, and pulmonary functional characteristics of patients with pSS-ILD with and without progression. We found no significant association between progressive pSS-ILD and clinical and laboratory features, including age, smoking, SSDDI, organ involvement (salivary and lacrimal glands, lymphadenopathy, splenomegaly, arthritis, purpura, and autoimmune hepatitis), SGUS findings, or treatment regimen. Autoantibody profiles and levels of RF, IgG, C3, and C4 at baseline and the last follow-up CT, FVC, and DLCO were comparable between the two groups.

**Table 2:** Comparison of clinical, laboratory, and pulmonary characteristics between pSS patients with and without ILD progression.

	pSS-ILD with progression (n = 19)	pSS-ILD without progression (n = 20)	*P*-value
Age at the baseline CT, years	72.0 (62.0, 76.0)	68.0 (56.0, 77.7)	0.647^a^
Male, n (%)	3 (15.8)	4 (20)	1.000^b^
Interval between the baseline and last CT, years	3.73 (2.9, 10.0)	2.45 (1.1, 4.3)	0.054^a^
Body mass index, kg/m^2^	24.5 (22.6, 52.1)	22.1 (20.2, 26.0)	0.879^a^
USFR, ml/minute	0.06 (0.00, 0.20)	0.16 (0.04, 0.46)	0.079^a^
Smoking, n (%)	4 (21.1)	2 (10)	0.407^b^
Laboratory findings			
Positive anti-Ro60, n (%)	12 (63.1)	11 (55)	0.605^b^
Positive anti-Ro52, n (%)	11 (57.8)	14 (70)	0.564^b^
Positive anti-La/SSB, n (%)	2 (10.5)	3 (15)	1.000^b^
Rheumatoid factor, IU/ml	14 (7.5–19.5)	9 (7–31)	0.840^a^
IgG, mg/dL	1330 (1106.5–1794.25)	1510 (961.25–2703.0)	0.624^a^
LDH, mg/dL	236.5 (201.0–268.0)	223.5 (183.0–269.5)	0.784^a^
**Focus score**	1 (1, 2)	1 (1, 1.5)	0.493^a^
**Total SGUS OMERACT scores**	3 (0, 6.75)	6 (2.5, 8.0)	0.140^a^
Sjögren's Syndrome Disease Damage Index	4 (2,5)	4 (3, 5)	0.988^a^
Any Treatment for ILD, n (%)	10 (52.6)	8 (40)	0.527^b^
Rituximab	2 (10.5)	3 (15)	0.676^b^
Cyclophosphamide	5 (26.3)	4 (30)	0.716^b^
Mortality, n (%)	3 (15.8)	1 (5)	0.342^b^
FVC and DLCO at initial CT			
FVC, % predicted	82 (73.5, 95.5)	80 (62, 106)	0.851^a^
DLCO, % predicted	62 (54, 72)	67 (45.0, 75.5)	0.731^a^
FVC and DLCO at last CT			
FVC, % predicted	63 (60, 91)	81 (73.0, 109.0)	0.070^a^
DLCO, % predicted	60 (50, 68)	61.5 (45.7, 68.2)	0.988^a^
Relative changes in FVC, % predicted	− 11.8 (− 18.1, − 4.0)	4.7 (2.3, 12.3)	** < 0.001**^a^
Relative changes in DLCO, % predicted	2.7 (− 10.7, 8.2)	1.5 (− 8.9, 7.7)	0.760^a^

Values are expressed as median (Q1, Q3) unless otherwise stated. Intergroup comparisons were conducted using the ^a^Mann–Whitney U test and ^b^Fisher’s exact test. pSS: primary Sjogren’s syndrome, ILD: interstitial lung disease, CT: computed tomography, Unstimulated salivary flow rate, SGUS: salivary gland ultrasonography, OMERACT: Outcome Measures in Rheumatology Clinical Trials, SSDDI: Sjögren's Syndrome Disease Damage Index, FVC: Forced vital capacity, DLCO: Diffusing capacity for carbon monoxide.

Significant values are in bold.

**Figure.1:**
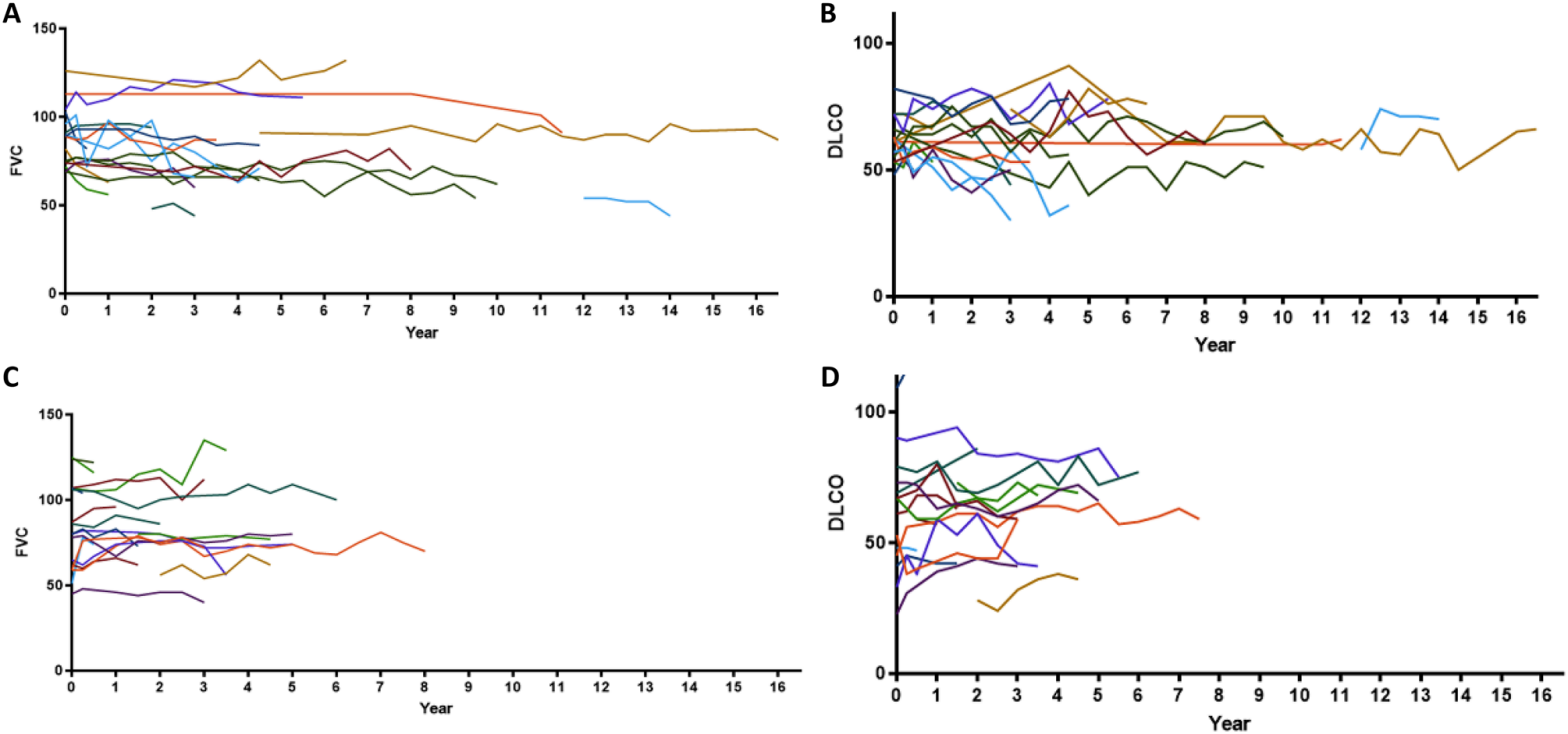
Changes in pulmonary function tests in patients with pSS-ILD during the follow-up period. Plots are shown for forced vital capacity (FVC) (**A**) and diffusing capacity for carbon monoxide (DLCO) (**B**) in pSS-ILD patients with progression and FVC (**C**) and DLCO (**D**) in those without progression.

Figure 1 shows longitudinal changes in FVC and DLCO in pSS-ILD with and without progression. Changes in FVC and DLCO were not monotonous in both groups. In progressors, FVC was significantly decreased at the last follow-up, compared with the baseline (*p* < 0.01, Wilcoxon signed-rank test), but DLCO did not show a significant change. Contrarily, no significant changes in FVC and DLCO were observed in non-progressors.

The relative change in FVC was -11.8% and 4.7% in progressors and non-progressors of pSS-ILD (*p* < 0.001), respectively, but the change in DLCO was comparable between the two groups (Table 2). On follow-up of PFTs, annual changes in FVC (-1.91%/year vs. 0%/year, *p* < 0.05) and DLCO (0.09%/year vs 0.81%/year, *p* = ns) also showed similar pattern (online supplemental Table S2).

### Comparisons of HRCT findings between progressive and non-progressive pSS-ILD

**Figure. 2:**
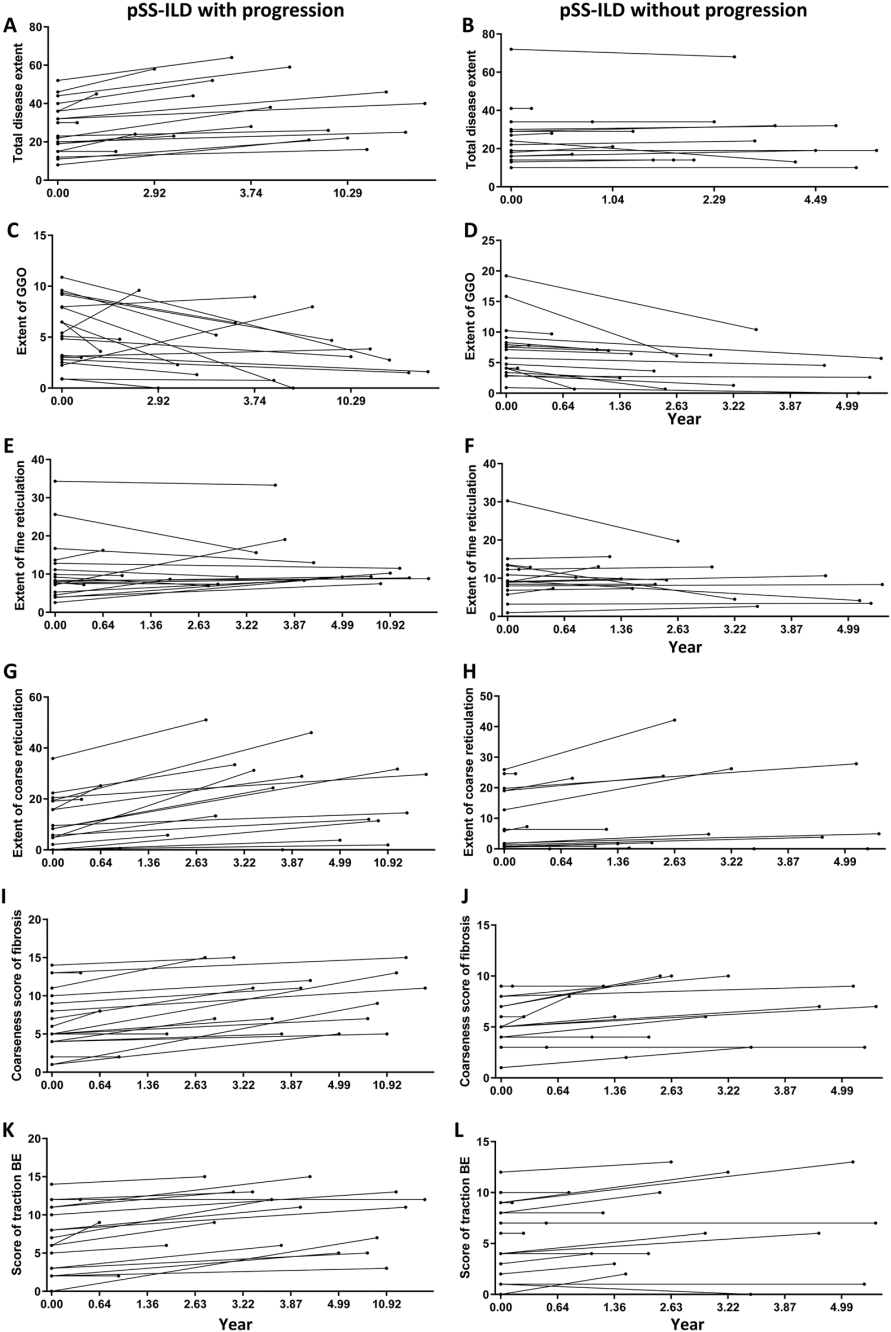
Changes in HRCT variables in patients with pSS-ILD with and without progression during the follow-up period. Plots are shown for the total extent (**A**, **B**) ground-glass opacities (GGO) (**C**, **D**), fine reticulation (**E**, **F**), coarse reticulations (**G**, **H**), coarseness score of fibrosis (**I**, **J**), and score of traction bronchiectasis (BE) (**K**, **L**). The left panel show patients with pSS-ILD with progression, and the right shows those without progression.

Figure 2 shows the changes in HRCT findings between progressive and non-progressive pSS-ILD. The total disease extent was increased only in progressors (*p* < 0.001; Wilcoxon signed-rank test), whereas a significant decrease in GGO (progressors *p* < 0.05; non-progressors *p* < 0.001) and an increase in coarse reticulation, coarseness score of fibrosis, and traction BE scores (progressors each *p* < 0.001; non-progressor each *p* < 0.01) were found in both groups over time.

Table 3 shows the HRCT characteristics of patients with pSS-ILD with and without progression. Nineteen (48.7%) patients with pSS-ILD had progression of ILD. The median follow-up CT interval was marginally longer in the progressive group than in the non-progressive group (3.73 vs. 2.45 years, *p* = ns). At baseline HRCT, UIP pattern was significantly more prevalent in patients with progressive than non-progressive pSS-ILD (32% vs. 5%, *p* < 0.05). Multivariate logistic regression analysis showed that the follow-up duration (OR, 1.403 [95% CI 1.055–1.868]) and UIP pattern on HRCT (OR 15.237 [95% CI (1.382–168.029]) were independently associated with the progression of ILD in pSS patients (Supplementary Table S3). However, none of the CT variables for lung fibrosis at baseline showed a significant association with ILD progression. On evaluation of the last follow-up HRCT, patients with progressive pSS-ILD had higher total disease extent (30.0% vs. 22.5%, *p* < 0.05), coarse reticulation (19.8% vs. 4.8%, *p* < 0.05), and coarseness score of fibrosis (9.0% vs. 6.5%, *p* < 0.05) than those with non-progressive pSS-ILD.

**Table 3:** Comparison of HRCT findings between pSS patients with and without ILD progression.

	Total pSS-ILD (n = 39)	pSS-ILD with progression (n = 19)	pSS-ILD without progression (n = 20)	*P*-value
Baseline CT findings				
UIP pattern, n (%)	7 (17.9)	6 (31.6)	1 (5.0)	**0.044**^**a**^
OP pattern, n (%)	3 (7.7)	0 (0.0)	3 (15.0)	0.231^**a**^
Total disease extent	23.0 (15.5, 34.0)	23.0 (15, 36)	23.0 (15.5, 31.0)	1.000^b^
Extent of GGO	5.4 (3.1, 8.1)	5.1 (2.8,7.9)	6.4 (3.9, 8.5)	0.391^b^
Extent of fine reticulation	8.9 (6.3, 12.4)	8.0 (6.2, 11.9)	9.1 (7.4, 12.3)	0.708^b^
Extent of coarse reticulation	5.34 (0.1, 19.0)	8.3 (1.1–71.5)	1.7 (0.2, 1.59)	0.385^b^
Presence of HC, n (%)	18 (46.1)	11 (57.9)	7 (35.0)	0.248^**a**^
Coarseness score of fibrosis (0–15)	5 (4, 8)	5 (4, 10)	5 (3.7–7.2)	0.343^b^
Scores of traction bronchiectasis (0–15)	7 (3, 9.5)	7 (3, 11)	6.5 (2.7–9.0)	0.391^b^
Last follow-up CT findings				
Total disease extent	28.0 (19.0, 40.5)	30 (23.0, 46.0)	22.5 (14.0, 32.5)	**0.036**^b^
Extent of GGO	3.8 (1.5, 6.4)	3.01 (1.5, 5.2)	5.13 (2.2, 7.0)	0.327^b^
Extent of fine reticulation	9.3 (7.34, 12.9)	9.24 (8.3, 12.9)	9.52 (4.4, 12.8)	0.418^b^
Extent of coarse reticulation	11.7 (1.8, 26.6)	19.8 (5.7, 31.2)	4.8 (0.28, 23.8)	**0.025**^b^
Presence of HC, n (%)	27 (69.2)	15 (78.9)	12 (60.0)	0.476^**a**^
Coarseness score of fibrosis (0–15)	7 (5, 10.5)	9 (5, 13)	6.5 (3.75, 9.0)	**0.031**^b^
Scores of traction bronchiectasis (0–15)	8 (5, 12)	11 (6, 13)	6.5 (3.7, 10.0)	0.066^b^
Absolute changes of HRCT scores between baseline and last follow-up				
Total disease extent	3 (0–9)	9 (4–12)	0 (0–2.0)	** < 0.001**^b^
Extent of GGO	-1.2 (-3.4, -0.2)	-1.3 (-4.1,-0.0)	-1.1 (-3.4,-0.5)	0.893^b^
Extent of fine reticulation	0.3 (-1.1, 1.6)	0.4 (-0.1, 3.5)	0.2 (-0.8, 0.9)	0.425^b^
Extent of coarse reticulation	3.7 (0.6–11.2)	9.12 (3.6, 15.1)	1.25 (0, 4.2)	**0.003**^b^
Coarseness score of fibrosis (0–15)	2 (0, 3)	2 (1, 4)	1 (0, 2)	0.189^b^
Scores of traction bronchiectasis (0–15)	0 (1, 3)	3 (1, 3)	0.5 (0, 2)	**0.011**^b^
Annual changes of HRCT scores				
△Total disease extent/year	0.7 (0, 2.6)	2.4 (0.5, 3.7)	0.0 (0.0, 0.7)	**0.001**^b^
△Extent of GGO/year	-0.4 (-0.9, -0.5)	-0.2 (-0.7, -0.0)	-0.5 (-1.1, -0.2)	0.118^b^
△Extent of fine reticulation/year	0.0 (-0.6, 3.5)	0.0 (-0.4, 0.6)	0.0 (-0.9, 0.4)	0.708^b^
△Extent of coarse reticulation/year	0.4 (0.0, 0.7)	1.6 (0.6, 3.9)	0.5 (0.0, 2.0)	**0.021**^b^
△Coarseness score of fibrosis/year	0.4 (0.0, 0.7)	0.5 (0.1, 0.7)	0.3 (0.0, 0.6)	0.461^b^
△Scores of traction bronchiectasis/year	0.3 (0.0, 0.7)	0.5 (0.1, 0.7)	0.1 (0.0, 0.7)	0.223^b^

Values are expressed as median (Q1, Q3) unless otherwise stated. Intergroup comparisons were conducted using ^a^Fisher’s exact test and ^b^Mann–Whitney U test. pSS: primary Sjogren’s syndrome, ILD: interstitial lung disease, HRCT: high resolution computed tomography, UIP: usual interstitial pneumonia, OP: organizing pneumonia, GGO: ground-glass opacities, HC: honeycombing.

Significant values are in bold.

Both absolute and annual changes in total disease extent (9% vs. 0%, *p* < 0.001) (2.4%/year vs. 0%/year, *p* < 0.001) and coarse reticulation (9.12% vs. 1.25%, *p* < 0.01) (1.66%/year vs. 0.55/year, *p* < 0.05) were significantly higher in progressive than non-progressive group of patients with pSS-ILD. The changes in the extent of GGO and fine reticulations were not statistically significant. Although the absolute change in traction BE score was higher in patients with progressive pSS-ILD, the annual change in traction BE score was not statistically significant.

### Treatment response in pSS-ILD based on HRCT scores

Eighteen patients with pSS-ILD were administered any treatment (11 patients with progressive pSS-ILD and seven with non-progressive pSS-ILD). Treatment regimen of cyclophosphamide included 7,501,000 mg intravenous infusions every 1 month for six doses and corticosteroids, and that of rituximab included two 1 g intravenous infusions separated by 2 weeks and corticosteroids. Three patients with progressive pSS-ILD received maintenance therapy with azathioprine after cyclophosphamide therapy. Other patients did not receive maintenance therapy with oral immunosuppressive agents other than glucocorticoids. Two patients each in the progressor and non-progressor groups received glucocorticoid monotherapy, and the duration of high-dose glucocorticoid (≥ 0.5 mg/kg/day) was less than 1 month.

**Figure. 3:**
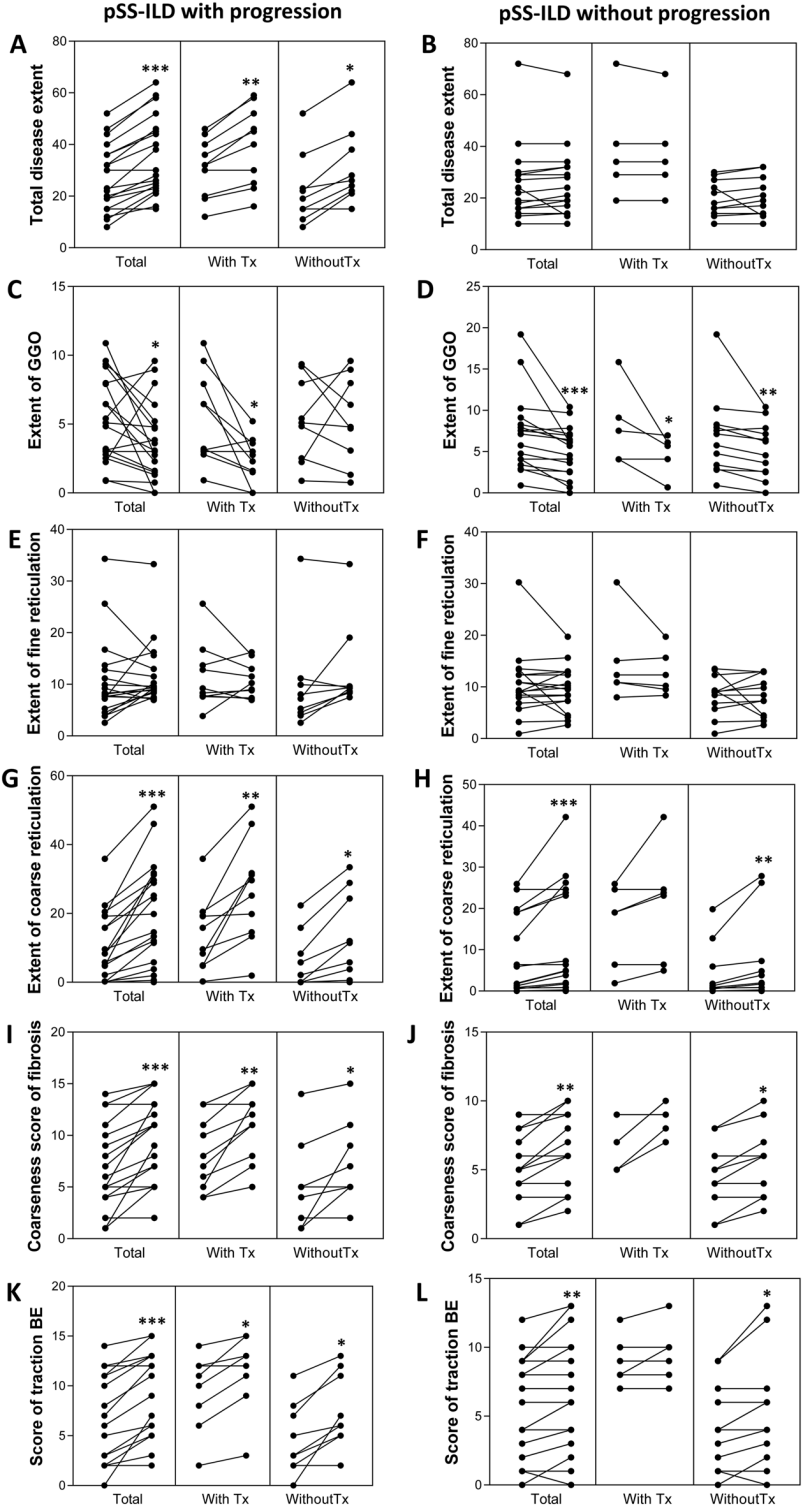
Changes in HRCT variables in pSS-ILD patients according to progression and treatment during the follow-up period. Plots are shown for the total extent (**A**, **B**) of ground glass opacities (GGO) (**C**, **D**), fine reticulation (**E**, **F**), coarse reticulations (**G**, **H**), coarseness score of fibrosis (**I**, **J**), and score of traction bronchiectasis (BE) (**K**, **L**). The left panels show patients with pSS-ILD with progression, and the right panel shows those without progression. *p-value < 0.05, **p-value < 0.01, ***p-value < 0.001 based on the Wilcoxon signed-rank test.

Figure 3 shows changes on HRCT in progressive and non-progressive pSS-ILD with and without treatment. In progressive pSS-ILD, total disease extent, coarse reticulation, coarseness score of fibrosis, and traction BE scores were significantly increased at the last follow-up compared to the baseline, irrespective of treatment (all *p* < 0.05, Wilcoxon signed-rank test). However, the extent of GGO was significantly decreased in progressors treated for pSS-ILD. In non-progressors, GGO was significantly decreased at the last follow-up compared to that at the baseline, irrespective of treatment (all *p* < 0.05). However, coarse reticulation, coarseness score of fibrosis, and traction BE scores were significantly decreased only in non-progressors who did not receive treatment for pSS-ILD (all *p* < 0.05).

**Figure. 4:**
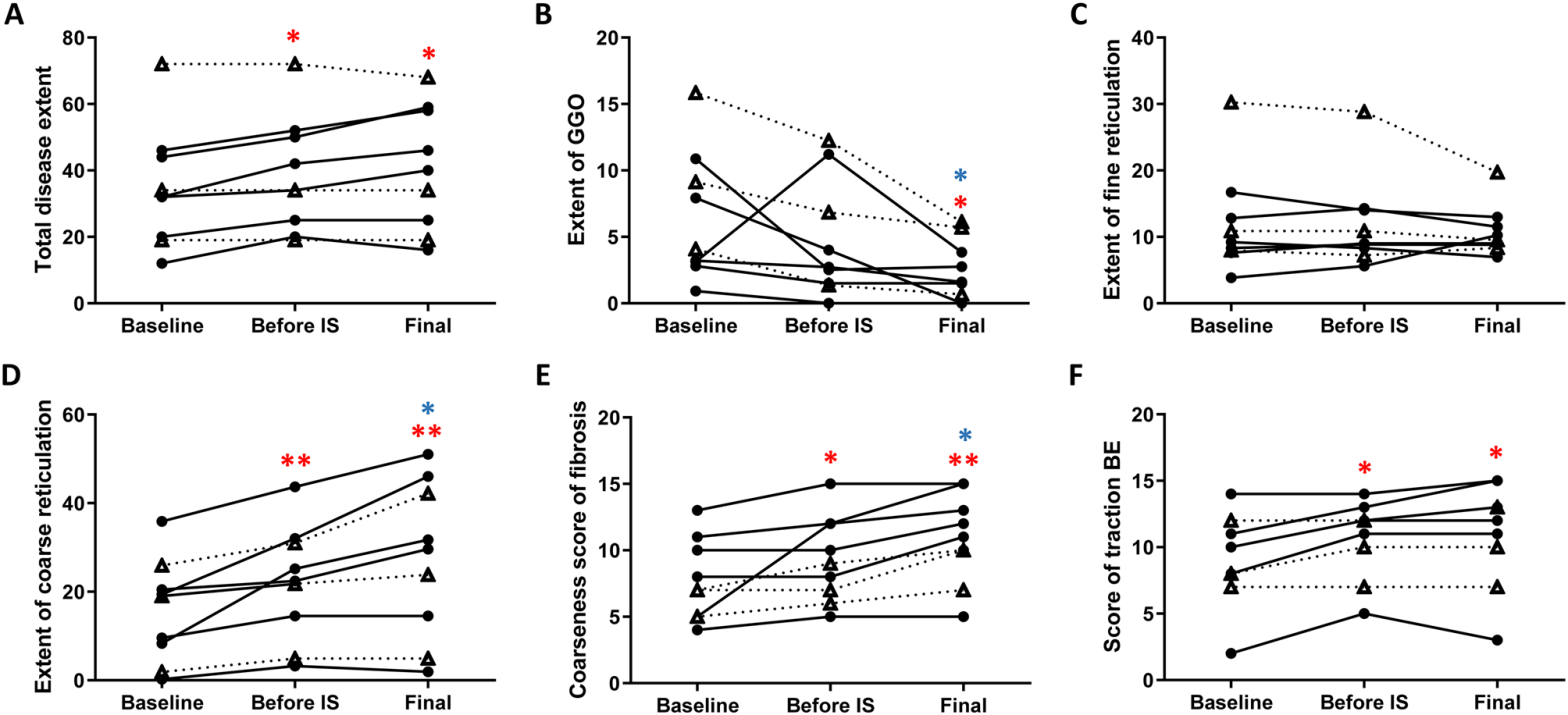
Changes in HRCT variables in patients with pSS-ILD before and after treatment. Plots are shown for the total extent (**A**) ground-glass opacities (GGO) (**B**), fine reticulation (**C**), coarse reticulations (**D**), coarseness score of fibrosis (**E**), and score of traction bronchiectasis (BE). Blank triangles and dotted lines indicate pSS-ILD patients without progression, and filled circles and solid lines represent patients with progression. *p-value < 0.05, **p-value < 0.01, based on the Wilcoxon signed-rank test, Red asterisk: compared to baseline CT, Blue asterisk: compared to before immunosupressants (IS).

Among them, 9 patients (6 with progressive pSS-ILD and 3 with non-progressive pSS-ILD) underwent HRCT scans prior to initiating immunosuppressive agents (rituximab + glucocorticoids [n = 3], cyclophosphamide + glucocorticoids [n = 5], and glucocorticoids [n = 1]). The median interval between baseline and follow-up HRCT prior to starting the immunosuppressants was 3.35 (1.57, 7.28) years, and the interval between HRCT before treatment and the last follow-up exam after starting treatment was 1.90 (1.00, 3.08) years. The total disease extent and traction BE score increased before starting the immunosuppressants (each *p* < 0.05, respectively), which remained stable at the last follow-up CT scan after treatment (Fig. 4). The extent of GGO was significantly decreased at the last follow-up CT compared to that at baseline (*p* < 0.05) and before treatment (*p* < 0.05). In contrast, the extent of coarse reticulation and coarseness score of fibrosis increased before starting immunosuppressants compared to baseline (*p* < 0.01 and *p* < 0.05, respectively), and were also significantly increased even after treatment compared to those before treatment (*each p* < 0.05). When six patients with progression were analyzed separately, similar trends were observed. The extent of GGO was significantly decreased at the last follow-up CT compared to that at the baseline (*p* < 0.05; Wilcoxon signed-rank test). The total disease extent and coarseness reticulation were increased before administering immunosuppressants (each *p* < 0.05), and the coarseness scores were increased even after treatment, compared to those before treatment, with marginal significance (*p* = 0.066). Meanwhile, three patients without progression showed non-significant changes on HRCT\ before and after immunosuppressant administration.

### Mortality

The median follow-up periods of patients with and without pSS-ILD were 4.17 (2.33, 5.57) and 4.57 (2.93, 5.46) years, respectively. During the follow-up period, all the patients without pSS-ILD survived and 4 (10.26%) with pSS-ILD died (*p* = 0.01). The causes of death were acute exacerbation of ILD (n = 2), acute exacerbation of ILD or pneumonia (n = 1), and pulmonary non-Hodgkin's lymphoma (n = 1).

## Discussion

In the present study, we did not identify newly developed ILD in patients with pSS without ILD at the baseline during the follow-up period of 2.8 years. In patients with pSS-ILD, the extent of coarse reticulation and traction BE were increased, whereas the extent of GGO was decreased during the follow-up period of 3.2 years. Progression occurred in 48.7% of patients with pSS-ILD, with a slowly worsening course. Low DLCO and high LDH were independent risk factors for the presence of pSS-ILD at the baseline, and the UIP pattern on HRCT and longer follow-up periods were associated with the progression of pSS-ILD. Our longitudinal HRCT analysis showed that the extent of pulmonary fibrosis increased, even after administering immunosuppressive agents.

Interstitial lung disease occurs in 10–20% of pSS patients, which is the most common pulmonary manifestation^3^. However, data on the long-term course of ILD are limited. In this study, we observed a variable time of ILD onset in patients with pSS. The diagnosis of ILD preceded the clinical diagnosis of pSS in 33.3% of our patients, with a median delay of 1.5 years. Similarly, a previous study by Roca et al. reported that ILD preceded pSS onset in 25% patients with a median delay of 15 months. They also showed that ILD developed after pSS onset in 45% of the patients^5^. Another cohort study demonstrated that among patients with pSS without prior ILD (n = 105), the cumulative incidence of pSS-ILD was 10% at one year after the initial diagnosis of pSS and was increased by 20% at 5 years^20^. In contrast, ILD was not observed in our study patients who had no ILD at baseline CT during the median follow-up period of 2.8 years. We believe that this discrepancy can be explained by the modality of diagnosis and follow-up of ILD. We enrolled patients who underwent at least two CT evaluations at baseline and follow-up with a minimum interval of six months, and the diagnosis of ILD was based on HRCT findings. Therefore, we could have included patients with pSS with preclinical ILD or with a minimal extent of early ILD. In previous studies, ILD was diagnosed not only with HRCT but also with chest radiography and PFTs, and patients with preclinical or early ILD were excluded^20^. Additionally, the median length of follow-up was 9.2 years in a previous cohort study, which is much longer than that in our study. Although the follow-up duration in our study was relatively short (median, 2.8 years), we can suggest that routine surveillance using chest CT is not necessary for 2–3 years in pSS patients who have no ILD at baseline evaluation. On the contrary, development of systemic sclerosis (SSc) -associated ILD usually observed within the early in the course of SSc. Therefore, PFTs can be useful every 4–6 months in the first 3 years after an SSc diagnosis for early detection and monitoring progression^21^. Different strategies for management of pSS-ILD and SSc-ILD can be required due to the different course of ILD. Further long-term studies are required to investigate the occurrence of ILD in patients of pSS.

We found that the CT extent of total lung disease and pulmonary fibrosis was significantly increased, and the extent of GGO was decreased on follow-up in our patients with pSS-ILD. When patients were divided into two groups, progressive and non-progressive pSS-ILD groups, the extent of coarse reticulation increased significantly in the progressors. On HRCT, GGO not only suggests the presence of inflammation but also indicates the presence of fibrosis below the resolution of CT, which can be referred to as early ILD. We believe that the progression of pulmonary fibrosis shown in our pSS patients corresponds well with the evolving CT findings from GGO to reticulation. According to recent guidelines for progressive pulmonary fibrosis (PPF)^22^, the pattern of progression is variable in ILD other than idiopathic pulmonary fibrosis and may include the evolution of GGO to a reticular abnormality. Our study shows that HRCT is a crucial modality that can determine the progression of lung fibrosis in patients with pSS, in addition to the initial screening evaluation for ILD. Despite treatment with glucocorticoids and immunosuppressive agents, patients with progressive pSS-ILD showed an increase in the extent of coarse reticulation. In contrast, non-progressors who did not receive treatment showed an increased extent of coarse reticulation, but those who did showed no significant changes in coarse reticulation. These findings suggest that in some patients, inflammation was improved and fibrosis progression decreased in response to the anti-inflammatory treatment; however, in others, there was no response to current immunosuppressive agents and fibrosis continued to progress. Based on our results, not only immune modulatory treatment but also other treatment options for progressive pSS-ILD, such as anti-fibrotic agents, should be considered. Indeed, further prospective studies are needed to reveal the biomarkers which can predict progressive pSS-ILD unresponsive to immunosuppressive treatment.

Although FVC is highly reproducible and DLCO is highly sensitive in predicting the presence of ILD^3^, our long-term follow-up of FVC and DLCO showed no significant changes in total patients with pSS-ILD. This finding can be attributed to the missing FVC and DLCO data in patients with severe pSS-ILD. In particular, PFTs could not be performed in four mortality cases. As reduced DLCO is a more common abnormality in pSS-ILD than in other PFT parameters^23^, our study also showed a comparable baseline FVC and a decline in DLCO in pSS patients with ILD compared to those without ILD at baseline. However, in patients with progressive pSS-ILD, a decrease in FVC was the dominant finding, but the changes in DLCO were comparable between progressors and non-progressors. Although HRCT is a highly sensitive tool for evaluating the presence and severity of pSS-ILD, serial PFTs with DLCO are likely to aid in identifying preclinical diseases and guiding the appropriate time for CT follow-up^7^.

Consistent with previous studies^3,24^, our results suggest that LDH and decreased DLCO at baseline are independent risk factors for ILD. However, these factors were not associated with pSS-ILD progression. Previous studies have reported that the UIP pattern on CT was an independent predictor of disease progression and mortality in patients with pSS-ILD^6,11^. Similarly, our results indicate that the UIP pattern on HRCT is associated with progressive pSS-ILD, which suggests that more careful monitoring is essential in patients with pSS-ILD and UIP patterns in terms of PPF. Moreover, a longer follow-up duration was also associated with progression of pSS-ILD, which reflects the gradual deteriorating course of pSS-ILD over time and the need for observation and proper monitoring of these patients.

Our study has some limitations. This retrospective observational study was conducted at a single center with a relatively small number of patients with pSS-ILD. As HRCT was not performed annually and consensus guidelines for pSS-ILD are lacking, intervals between two CT scans were not consistent. Although there were no significant differences in CT intervals between the pSS-ILD groups with and without progression, one major limitation of our study is the different time points of the last CT scan. A large prospective cohort with long-term follow-up is required to verify the characteristics of disease progression and identify the risk factors for poor outcomes to provide insights into the management of pSS-ILD. Although several studies have shown that a six-min walk distance is an independent predictor of mortality in ILD, data on the 6-min walk distance were missing.

In conclusion, in pSS patients with no ILD during baseline evaluation, no newly developed ILD was identified during the follow-up over 2 years. Progression occurred in approximately half of the pSS-ILD patients with slow gradual deterioration. Further, the extent of fibrosis increased even after anti-inflammatory treatment.

## Supplementary Information

Supplementary Information.

## Data Availability

Data are available on reasonable request. The data that support the findings of this study are available from the corresponding author on reasonable request.
