# Supplementary material for: Clinical course and risk factors for development and progression of interstitial lung disease in primary Sjögren’s syndrome
Source: Sci Rep. 2023 Jun 6;13:9189. doi: 10.1038/s41598-023-35608-4 (PMC10244322; doi:10.1038/s41598-023-35608-4)
Supplement: Supplementary file 1 — Supplementary Information. [file 41598_2023_35608_MOESM1_ESM.docx]

**Supplementary Material**

**Clinical course and risk factors for development and progression of interstitial lung disease in primary Sjögren’s syndrome**

**Kyung-Ann Lee^a^**^†^**, Bo Da Nam^b^**^†^**, Jung Hwa Hwang^b^, and Hyun-Sook Kim^a*^**

^a^Division of Rheumatology, Department of Internal Medicine, Soonchunhyang University Seoul Hospital, Soonchunhyang University School of Medicine, Seoul, South Korea

^b^Department of Radiology, Soonchunhyang University Seoul Hospital, Soonchunhyang University School of Medicine, Seoul, South Korea

^†^These authors contributed equally to this work.

**Supplementary Data S1. Definition for HRCT variables**

1. Total disease extent

The extent of individual CT features (GGOs, reticulations, and honeycombing) was estimated on each of the five sections (between 0% and 100%, censored at 5%) and the total extent of fibrosis was the mean score of each individual CT features.

1. Extent of individual features

Individual HRCT features included GGO, reticulation, and honeycombing, based upon the Fleischner Society’s glossary of terms for thoracic imaging [^1^](#_ENREF_1). (i) GGO (increased parenchymal density with preservation of bronchial and vascular markings) (ii) fine reticulation (including intralobular septal thickening—delicate criss-crossing linear opacities separated by less than a quarter of the diameter of a pulmonary lobule with or without GGOs; (iii) coarse reticulation (linear opacities than were thicker than fine reticulation and separated by more than a quarter of the diameter of a pulmonary lobule) [^2^](#_ENREF_2). The presence and relative proportion of each feature were analyzed in each section. Besides, the presence of honeycombing was recorded and the extent of honeycombing was included in the extent of coarse reticulation. This is because many previous studies have shown that it is difficult to distinguish honeycombing with coarse reticulation and inter-observer agreement is low [^3^](#_ENREF_3)^,^[^4^](#_ENREF_4).

1. Coarseness of fibrosis

The coarseness of fibrosis was quantified in each section as follows: grade 0 = GGO alone; grade 1 = fine reticulation; grade 2 = coarse reticulation or microcystic honeycombing; grade 3 = macrocytic honeycombing. The total coarseness score was the summed score of all five levels (range, 0-15). The coarseness score was adjusted to a five-level score in patients with no disease in one or more CT sections as previous study ^[5](#_ENREF_5)^.

1. Severity of traction bronchiectasis

Traction bronchiectasis was scored in each section with a four-point scale as follows: 0 = none, 1 = mild (limited to subpleural 1/3), 2 = moderate (subpleural 2/3), 3 = severe ^[2](#_ENREF_2)^. The total traction bronchiectasis score was the summed score for all five levels (range, 0-15).

1. Diagnosis of HRCT pattern

The categorization of HRCT pattern was based on 2013 American Thoracic Society classification of idiopathic interstitial pneumonias as follows: 1) usual interstitial pneumonia (UIP) pattern; 2) nonspecific interstitial pneumonia (NSIP) pattern; 3) organizing pneumonia (OP) pattern; 4) lymphocytic interstitial pneumonia (LIP) pattern [^6^](#_ENREF_6).

**References**

1. Hansell, D. M. *et al.* Fleischner Society: glossary of terms for thoracic imaging. *Radiology* **246**, 697-722 (2008).

2. Edey, A. J. *et al.* Fibrotic idiopathic interstitial pneumonias: HRCT findings that predict mortality. *European radiology* **21**, 1586-1593 (2011).

3. Walsh, S. L., Calandriello, L., Sverzellati, N., Wells, A. U. & Hansell, D. M. Interobserver agreement for the ATS/ERS/JRS/ALAT criteria for a UIP pattern on CT. *Thorax* **71**, 45-51 (2016).

4. Watadani, T. *et al.* Interobserver variability in the CT assessment of honeycombing in the lungs. *Radiology* **266**, 936-944 (2013).

5. Goh, N. S. *et al.* Interstitial lung disease in systemic sclerosis: a simple staging system. *American journal of respiratory and critical care medicine* **177**, 1248-1254 (2008).

6. Travis, W. D. *et al.* An official American Thoracic Society/European Respiratory Society statement: Update of the international multidisciplinary classification of the idiopathic interstitial pneumonias. *American journal of respiratory and critical care medicine* **188**, 733-748 (2013).

**Supplementary Figure S1. Flow diagram of the study design and patient enrolment.**

**
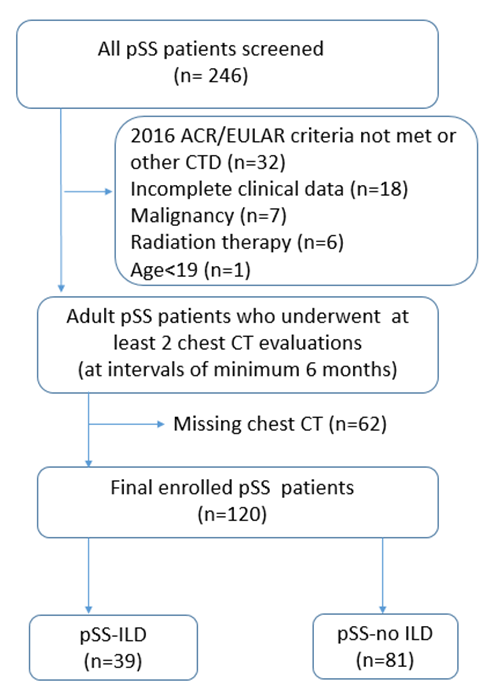
**

pSS: primary Sjogren’s syndrome, ACR/EULAR: American College of Rheumatology/ European League Against Rheumatism, CTD: connective tissue disease, CT: computed tomography

**Supplementary Table S1.** Univariate and multivariate logistic regression analyses of presence of ILD in pSS patients

| **Factors** | **Univariate analysis** | | **Multivariate analysis** | |
| --- | --- | --- | --- | --- |
|  | OR (95% CI) | P-value | OR (95% CI) | P-value |
| Age | 1.087 (1.044-1.131) | <0.001 |  |  |
| Male | 2.734 (0.852-8.778) | 0.091 |  |  |
| Smoking | 14.545 (1.685-125.558) | 0.015 |  |  |
| Levels of Anti-Ro52 | 1.296 (0.987-1.703) | 0.063 |  |  |
| Positive anti-Ro52 | 2.071 (0.931-4.607) | 0.074 |  |  |
| LDH | 1.018 (1.008-1.028) | <0.001 | 1.012 (1.000-1.024) | 0.038 |
| FVC at baseline | 1.000 (0.989-1.012) | 0.969 |  |  |
| DLCO at baseline | 0.911 (0.875-0.949) | <0.001 | 0.922 (0.886-0.961) | <0.001 |

ILD: interstitial lung disease, pSS: primary Sjögren’s syndrome, LDH: lactate dehydrogenase, FVC: Forced vital capacity, DLCO: Diffusing capacity for carbon monoxide

**Supplementary Table S2.** Annual changes of FVC, and DLCO in patients with pSS-ILD

|  | **Total pSS-ILD (n=39)** | **pSS-ILD progression (n=19)** | **pSS-ILD-stable/improvement (n=20)** | **P-value** |
| --- | --- | --- | --- | --- |
| **FVC, % predicted** |  |  |  |  |
| Initial FVC | 81 (68, 103.25) | 89 (73, 93.5) | 80 (59, 87) | 0.665 |
| Last FVC | 74 (62, 94.5) | 70 (61, 87) | 74 (70, 96) | 0.191 |
| Intervals between initial and last FVC, years | 3.1 (1.5, 5.3) | 4.2 (2.1, 7.5) | 3.0 (1.7, 4.8) | 0.191 |
| △FVC/year | -1.17 (-3.2, 0.89) | -1.91 (-4.4, -0.53) | 0.0 (-1.38, 1.81) | 0.021 |
| **DLCO, % predicted** |  |  |  |  |
| Initial DLCO | 61.0 (53, 72) | 61.0 (56.5, 69.0) | 61.0 (45, 73) | 0.940 |
| Last DLCO | 59.5 (50.0, 69.75) | 60.0 (50.5, 68.0) | 59.0 (47, 69.0) | 0.802 |
| Intervals between initial and last DLCO, years | 3.1 (1.7, 5.1) | 3.39 (1.97, 6.36) | 3.03 (1.75, 4.81) | 0.594 |
| △DLCO/year | 0.21 (-1.45, 1.59) | 0.09 (-2.47, 0.76) | 0.81 (-1.14, 2.3) | 0.100 |

Values are expressed as median (Q1, Q3). FVC: Forced vital capacity, DLCO: Diffusing capacity for carbon monoxide, ILD: interstitial lung disease, pSS: primary Sjögren’s syndrome,

| **Factors** | **Univariate analysis** | | **Multivariate analysis** | |
| --- | --- | --- | --- | --- |
|  | OR (95% CI) | P-value | OR (95% CI) | P-value |
| Follow-up duration, years | 1.298 (1.017-1.657) | 0.036 | 1.403 (1.055-1.868) | 0.020 |
| UIP pattern on HRCT | 8.769 (0.942, 81.671) | 0.057 | 15.237 (1.382-168.029) | 0.026 |
| Smoking | 2.400 (0.385-14.968) | 0.349 |  |  |
| Levels of Anti-Ro52 | 0.871 (0.555-1.367) | 0.547 |  |  |
| LDH | 1.001 (0.989-1.013) | 0.846 |  |  |
| FVC at baseline | 1.005 (0.972-1.040) | 0.750 |  |  |
| DLCO at baseline | 0.997 (0.957-1.040) | 0.895 |  |  |
| Total disease extent at baseline CT | 1.007 (0.959-1.058) | 0.771 |  |  |
| Extent of coarse reticulation at baseline CT | 1.031 (0.963-1.104) | 0.378 |  |  |

**Supplementary Table S3.** Univariate and multivariate logistic regression analyses of ILD progression in pSS

ILD: interstitial lung disease, pSS: primary Sjögren’s syndrome, UIP: usual interstitial pneumonia; HRCT: high-resolution computed tomography computed tomography, LDH: lactate dehydrogenase, FVC: Forced vital capacity, DLCO: Diffusing capacity for carbon monoxide
